# Electromagnetically Induced Transparency in a GaAs Coupled Quantum Dot-Ring

**DOI:** 10.3390/nano15181455

**Published:** 2025-09-22

**Authors:** R. V. H. Hahn, A. S. Giraldo-Neira, J. A. Vinasco, J. A. Gil-Corrales, A. L. Morales, C. A. Duque

**Affiliations:** 1 Departamento de Electrónica y Tecnología de Computadores, Facultad de Ciencias, Universidad de Granada, 18071 Granada, Spain; hahn@ugr.es; 2 Grupo de Materia Condensada-UdeA, Instituto de Física, Facultad de Ciencias Exactas y Naturales, Universidad de Antioquia UdeA, Calle 70 No. 52-21, Medellín 050010, Colombia; anna.giraldo@udea.edu.co (A.S.G.-N.); alvaro.morales@udea.edu.co (A.L.M.); 3 Departamento de Ciencias Básicas, Facultad de Ingeniería y Administración, Universidad Nacional de Colombia Sede Palmira, Palmira 763533, Colombia; javinascos@unal.edu.co; 4 Instituto Tecnológico Metropolitano (ITM)-Institución Universitaria, Campus Fraternidad, Calle 73 No. 76A-354 Vía al Volador, Medellín 050034, Colombia; johngil1275@correo.itm.edu.co

**Keywords:** coupled quantum dot-ring, electronic states, electric field effects, magnetic field effects, optical absorption coefficient, electromagnetically induced transparency

## Abstract

In this work, the ground and low-lying excited states in a GaAs coupled quantum dot-ring embedded in an AlGaAs cylindrical matrix are computed under the assumption of a finite confinement potential and an axisymmetric model by means of the finite element method and the effective mass approximation. The electron energy levels are studied as functions of the intensity of externally applied electric and magnetic fields. Electromagnetically induced transparency in the ladder configuration and linear optical absorption coefficient are calculated thereupon. Our results suggest that magnetic fields are more suitable than electric fields for controlling the optical properties of this nanostructure. Also, we found that the system’s response, however, exhibits a striking asymmetry: while the electromagnetically induced transparency is unexpectedly quenched under positive electric fields due to vanishing dipole transition matrix elements, this limitation is completely overcome by a magnetic field. Its application not only restores optical transparency across the full range of electric field values but also drives substantially larger energy level shifts and clear Aharonov–Bohm oscillations, making it a far more robust tool for controlling the optical properties of confined electrons in dot-ring coupled heterostructures.

## 1. Introduction

Quantum nanostructures such as quantum dots [1,2,3,4], lens-shaped structures [5,6,7], and tetrapods [8] exhibit unique electronic and optoelectronic properties, which make them suitable for applications in multiple fields, from bioimaging [9] to photovoltaics [10]. Among these, quantum rings (QRs) [11,12,13] have attracted particular interest due to their unique electronic and optical characteristics, including Aharonov–Bohm oscillations and quantum interference effects [14,15], which open possibilities for their integration into optoelectronic devices and quantum information technologies [16,17,18].

During the characterization and fabrication process of such structures, it has been possible to experiment with various geometries [19,20,21,22], many of which are based on coupling structures. In recent years, coupled quantum dot-ring (CQDR) systems [23,24,25,26] have attracted significant attention due to their highly tunable electronic states, which can be modified by external factors such as electric and magnetic fields, hydrostatic pressure, and structural parameters [27]. This tunability makes CQDRs promising candidates for applications in optoelectronic devices, optical modulators [28], and quantum communication technologies [29]. These structures, formed by a QR coupled to a concentric quantum dot (QD), offer a versatile way to explore quantum interference phenomena and electromagnetically induced transparency (EIT).

CQDRs have also gained relevance due to their ability to host localized electronic states with versatile optical properties [25,27,30,31]. External perturbations on the structure, such as electric and magnetic fields, can substantially modify the electronic structure of these systems [32,33,34,35]. For example, the application of an electric field along the growth direction introduces Stark shifts and dipole moment variations [36,37,38]. This motivates continued studies that include these external perturbations.

Recent studies on the interaction between donors and structures have yielded interesting conclusions. Some have concluded that the interaction between the confined electron and the neutral donor center results in position-dependent modifications to transition energies and absorption properties [33,39,40]. Shallow donors add a layer of complexity to CQDRs due to their position within the structure [41,42], which plays a critical role in shaping electronic states and optical absorption spectra. Furthermore, recent studies on doubly ionized donors confined in CQDRs have provided information on the role of donor states and their interaction with external probes, such as hydrostatic pressure, electric fields, and temperature variations [27]. The present study explores the interaction between a shallow donor and a CQDR.

Some analytically known results have been performed concerning a three-dimensional electron gas under an applied magnetic field. When a magnetic field, *B*, parallel to the *z*-axis, is applied to a three-dimensional electron gas, the motion in the xy-plane is quantized into Landau levels with energy eigenvalues $E_{xy}=\hbar \;\omega_{B}\;\left(n+\frac{1}{2}\right)$, where *n* is a discrete quantum number, *ℏ* is the reduced Planck constant, and $\omega_{B}=\frac{e\;B}{m^{*}}$ is the cyclotron frequency. Here, $m^{*}$ is the effective mass and q=−e is the electron charge. Now, for a single QD, with parabolic confinement in the xy-plane [43] and a quantum well (QW) in the *z*-axis and under a “perpendicular” magnetic field applied in the *z*-direction [44], the system is rotationally symmetric relative to the *z*-axis, i.e., the QD retains its cylindrical symmetry. The total eigenenergies are given by $E_{xy}+E_{z}$. The *z*-axis eigenenergies are determined by the QW, i.e., $E_{z}=E_{m}$, where *m* is a natural number. For a parabolic confinement in the xy-plane, application of a “perpendicular” magnetic field along the *z*-axis changes the height of the energetic staircase and removes the degeneracy proportionally to *B*. This is the well-known Darwin–Fock [45,46,47,48,49] spectrum. In other words, the xy-plane eigenenergies become $E_{xy}=E_{n_{\rho },m_{l}}=\hbar \;\Omega \;(2\;n_{\rho }+|m_{l}\left|+1\right)\pm \hbar \;\omega_{B}\;m_{l}/2$, where the plus (+) sign corresponds to electrons and the minus (−) sign to holes and $\Omega^{2}=\omega_{B}^{2}/4+\omega_{pc}^{2}$. The energy scale pc stems from the “spatial” parabolic confinement in the xy-plane. On the other hand, $n_{\rho }$ is a natural number and $m_{l}$ is an integer. The states with $n_{\rho }=\;0,\;1,\;2,\;\ldots$ are often denoted as 1, 2, 3, …. The states with $m_{l}=0,\pm 1,\pm 2,\ldots$ are characterized as s,p,d, … [50,51,52].

In summary, the problem of a three-dimensional electron gas confined by a parabolic potential, for example, in the xy-plane, has an analytical solution that leads to the well-known Landau level spectrum. If, in addition, a magnetic field is applied perpendicular to the confinement plane, analytical solutions also arise, called Darwin–Fock spectra. In this case, the magnetic field breaks the degeneracy of the levels associated with equal absolute values of the magnetic quantum number. Energy spectra with similar behavior can be obtained in QRs, where it is possible to decouple the differential equations corresponding to motion in the plane from those describing the growth direction of the heterostructure. This is achieved, for example, by the effective mass approximation or by considering confinement in the growth direction as an infinite potential, the solution of which is also analytical. However, the problem we will address here differs substantially from these approximations, since the potential around the ring will be finite and the height of the structure in the growth direction will not be constant. For this reason, it is not possible to find an analytical solution, and it is necessary to resort to numerical procedures, such as solving differential equations using the finite element method (FEM).

Such facts motivate the present study of the energy levels of a GaAs/Al_0.3_Ga_0.7_As CQDR, along with a detailed analysis of the consequences of external factors, such as magnetic and electric fields, while identifying the optoelectronic properties of the heterostructure. Furthermore, this study examines the phenomenon of EIT, in which quantum interference suppresses absorption while maintaining significant nonlinear optical properties [53,54].

This paper is organized as follows: in Section 2, we describe the theoretical model, outlining the fundamental principles that govern the electronic and optical properties of CQDRs, along with a description of the basic theory. Section 3 presents and discusses our numerical results, highlighting the impact of external perturbations and structural parameters on the electronic states and optical responses. Finally, our main conclusions are provided in Section 4.

## 2. Theoretical Framework

In this work, we analyze the energy states and their associated wavefunctions in a CQDR structure under the influence of external electric and/or magnetic fields applied in the *z*-direction. To complete our study, we investigate optoelectronic properties via EIT in a ladder or cascade configuration and linear optical absorption.

**Figure 1:**
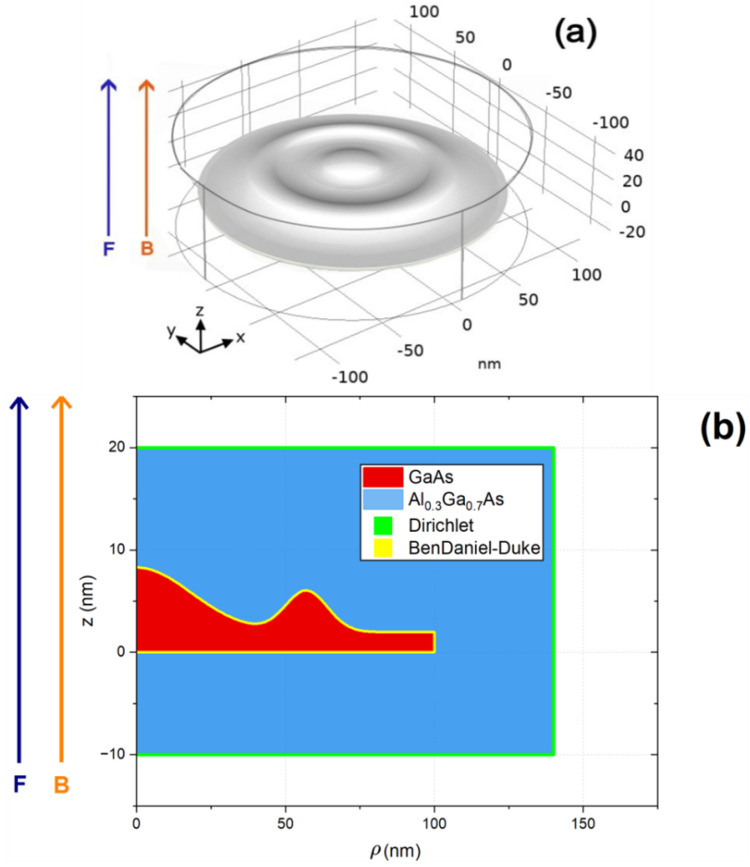
Schematic representation of the GaAs/${\mathrm{Al}}_{x}$G$a_{1-x}$As three-dimensional CQDR geometry (**a**), together with a zρ-plane slice of the CQDR and the cylindrical boundary for visualization of the main dimensions of the heterostructure (**b**). The reference system and the directions of the externally applied electric and magnetic field are also depicted.

Figure 1 presents a schematic view of the CQDR under study, together with the main dimensions of the heterostructure. A 3D view of the nanostructure is shown in Figure 1a, obtained by revolution around the *z*-axis of Figure 1b. A cross-section of the structure (the ϕ=0 plane) is shown in Figure 1b, where the two composing materials are easily identified, i.e., the GaAs and the ${\mathrm{Al}}_{x}$G$a_{1-x}$As. The latter displays a barrier-like behavior, while the former presents a well-like behavior. This configuration is suitable for building a 2D-axisymmetric model where azimuthal symmetry reduces the computational cost of numerically solving the three-dimensional Schrödinger equation. The geometry of the nanostructure is a variation of a theoretical function [33] that accurately reproduces an experimental profile of a GaAs CQDR, as obtained via the AFM technique by Somaschini [25]. To enhance the visibility of the ring’s influence, the height of the central region (dot) has been deliberately reduced by 65% compared to the original geometry. For the sake of clarity, the reference system and the directions of the external fields are also depicted. The CQDR is made of GaAs, whereas the cylindrical host matrix in which it is embedded is made of ${\mathrm{Al}}_{x}$G$a_{1-x}$As with x=0.3.

The mathematical function describing the height, h(ρ), as depicted in Figure 1a, is formally expressed as [27]:

(1) $$h\left(\rho \right)=h_{d}\exp\left[-{\left(\frac{\rho }{w_{d}}\right)}^{2}\right]+h_{r}\exp\left[-{\left(\frac{\rho -d_{r}}{w_{r}}\right)}^{2}\right]+w_{l}\;.$$

In this equation, $h_{d}$ represents the height of the central dot, $w_{d}$ denotes its characteristic width, $h_{r}$ signifies the height of the ring, $d_{r}$ corresponds to the radius of the ring, $w_{r}$ indicates the width of the ring, and $w_{l}$ establishes the baseline height. While the functional form of the nanostructure is inspired by Refs. [25,27], the specific values assigned to these parameters for the present investigation were chosen with the objective of enhancing the quantum ring effects, thereby fostering a strong interaction between the quantum dot and the quantum ring structures. For the specific nanostructure under investigation, the precise values assigned to these parameters are as follows: $h_{d}=6.3$ nm, $w_{d}=26$ nm, $h_{r}=4$ nm, $d_{r}=57$ nm, $w_{r}=10$ nm, and $w_{l}=2$ nm.

We note that the confinement along the *z*-axis down to 2 nm (approximately four times the GaAs lattice constant) places our use of the effective mass approximation (EMA) at the very limit of its theoretical validity. However, this choice is consistent with several experimental and theoretical studies on nanostructures of comparable dimensions (see, for instance, Refs. [55,56,57,58,59]). Moreover, the much larger radial confinement (on the order of 100 nm) provides additional support for the applicability of EMA in our case. Still, it is important to stress that our calculations operate at the edge of the EMA’s applicability. For systems with substantially smaller dimensions or different confinement parameters, more advanced atomistic models may be required. In this context, EMA should be understood as the lowest-order limit of $\vec{k}\cdot \vec{p}$ theory. Although convenient, it neglects band coupling and nonparabolic effects, and at confinement lengths of only a few nanometers (∼4 lattice constants), the envelope-function approximation becomes questionable. Therefore, the results presented here should be interpreted primarily as qualitative trends within the EMA framework, rather than as fully quantitative predictions on the atomistic scale.

Using the effective mass approximation and taking the assumption of parabolic conduction bands, the Schrödinger equation of an electron interacting with external electric and magnetic fields is written in cylindrical coordinates as follows:

(2) $$\left\{\frac{1}{2}\left[\left(-i\;\hbar \;\vec{\nabla }+e\;\vec{A}\right)\frac{1}{m_{j}^{*}\left(\vec{r}\right)}\left(-i\;\hbar \;\vec{\nabla }+e\;\vec{A}\right)\right]+e\;\vec{F}\cdot \vec{r}+V\left(\rho ,z\right)\right\}\;\Psi_{i}\left(\vec{r}\right)=E_{i}\;\Psi_{i}\left(\vec{r}\right)\;,$$

where *i* designates each of the quantized states, i.e., the value i=1 represents the ground state and i≥2 corresponds to the excited states. The *j*-index identifies each one of the two materials that make up the CQDR, and *e* is the absolute value of the electron charge. The values of the effective mass and V(ρ,z) depend on the region where the Schrödinger equation is solved, as shown in Figure 1a. The effective mass is considered to be position-independent within each material. The effective mass and the confining potential are taken for the calculations in the following way:

(3) $$V\left(\rho ,z\right)=\left\{\begin{array}{cc} 0, & (\rho ,z)\in \mathrm{CQDR}\;,\\ V_{0}, & (\rho ,z)\in \mathrm{cylindrical}\;\mathrm{matrix}\;,\\ \infty , & elsewhere\;, \end{array}\right.$$

and

(4) $$m_{j}^{*}\left(\vec{r}\right)=\left\{\begin{array}{cc} m_{GaAs}^{*}, & j=1\;,\\ m_{Al_{x}Ga_{1-x}As}^{*}, & j=2\;. \end{array}\right.$$

In this work, both electric and magnetic fields are applied along the *z*-direction, i.e., $\vec{F}=\left(F_{\rho },F_{\varphi },F_{z}\right)=\left(0,0,F_{z}\right)$ and $\vec{B}=\left(B_{\rho },B_{\varphi },B_{z}\right)=\left(0,0,B\right)$.

The gauge chosen for the description of the magnetic field effect entails the following conditions for the magnetic vector potential, $\vec{A}$: *(i) *$\vec{\nabla }\cdot \vec{A}=0$ and *(ii) *$\vec{A}=-\frac{1}{2}\vec{r}\times \vec{B}$. Under these two conditions, the Hamiltonian in Equation (2) takes the form:

(5) $$\left[-\frac{\hbar^{2}}{2\;m_{j}^{*}}\left(\frac{\partial^{2}}{\partial \;\rho^{2}}+\frac{1}{\rho }\frac{\partial }{\partial \;\rho }+\frac{1}{\rho^{2}}\frac{\partial^{2}}{\partial \;\varphi^{2}}\right)-\frac{e\;i\;\hbar \;B}{2\;m_{j}^{*}}\frac{\partial }{\partial \;\varphi }+\frac{e^{2}\;B^{2}\;\rho^{2}}{8\;m_{j}^{*}}+e\;F_{z}\;z+V\left(\rho ,z\right)\right]\Psi_{i}\left(\vec{r}\right)=E_{i}\Psi_{i}\left(\vec{r}\right)\;.$$

This Hamiltonian is separable, $H\left(\rho ,\varphi ,z\right)=H_{\varphi }\left(\varphi \right)+H_{\rho ,z}\left(\rho ,z\right)$, which allows to replace $\Psi_{i}\left(\vec{r}\right)=R_{i}\left(\rho ,z\right)\;e^{i\;l\;\varphi }$ in Equation (5) (here l=0,±1,±2,…). Taking the derivatives with respect to φ, the Schrödinger equation for the radial function $R_{i}\left(\rho ,z\right)$ can be written as:

(6) $$\left[-\frac{\hbar^{2}}{2\;m_{j}^{*}}\left(\frac{\partial^{2}}{\partial \;\rho^{2}}+\frac{1}{\rho }\frac{\partial }{\partial \;\rho }-\frac{l^{2}}{\rho^{2}}\right)+\frac{e\;\hbar \;B\;l}{2\;m_{j}^{*}}+\frac{e^{2}\;B^{2}\;\rho^{2}}{8\;m_{j}^{*}}+e\;F_{z}\;z+V\left(\rho ,z\right)\right]R_{i}\left(\rho ,z\right)=E_{i}^{\prime }\;R_{i}\left(\rho ,z\right)\;,$$

where $E_{i}^{\prime }$ are the eigenvalues associated with the 2D-wavefunctions $R_{i}$. The solution of Equation (6) was investigated using the FEM as implemented in the COMSOL-Multiphysics 6.2 software [60]. The boundary conditions were imposed in the following way: (i) BenDaniel–Duke boundary condition for wave function continuity on the core–shell interface, $R_{GaAs}=R_{Al_{x}Ga_{1-x}As}$, (ii) BenDaniel–Duke boundary condition for the wave function first derivative continuity: $\frac{1}{m_{GaAs}^{*}}\frac{\partial R_{GaAs}}{\partial X}=\frac{1}{m_{Al_{x}Ga_{1-x}As}^{*}}\frac{\partial R_{Al_{x}Ga_{1-x}As}}{\partial X}$ with X=ρ,z and (iii) Dirichlet boundary conditions on the outer surface of the embedding ${\mathrm{Al}}_{x}$G$a_{1-x}$As matrix: R(ρ,z)=0.

**Figure 2:**
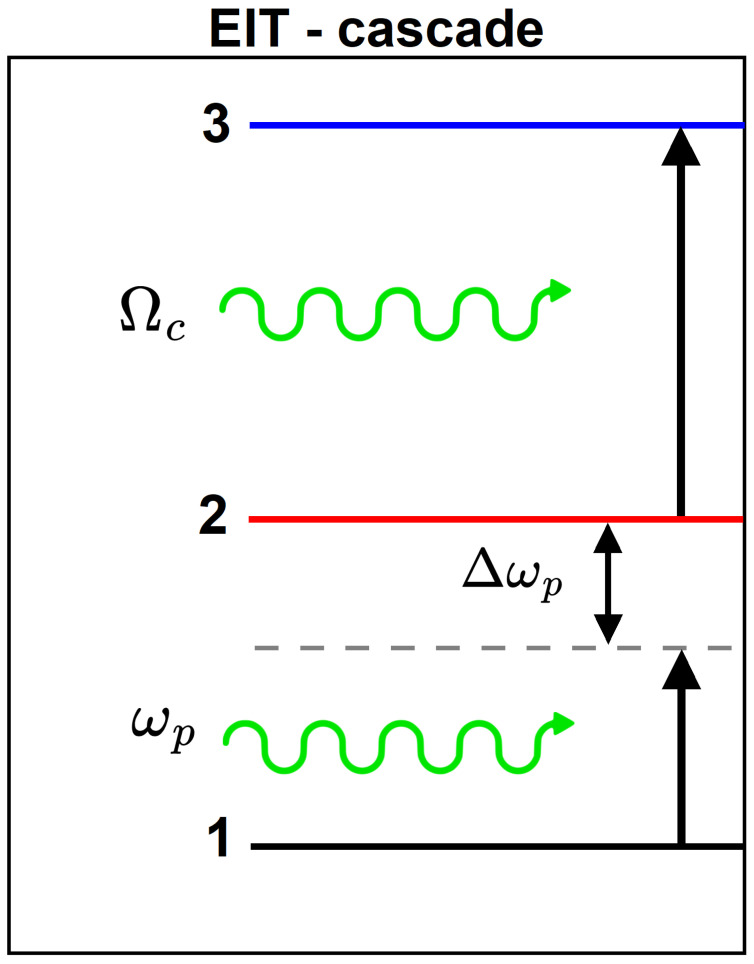
Schematic diagram of the three-level system in a ladder or cascade configuration interacting with control and probe electromagnetic fields of frequencies $\Omega_{c}$ and $\omega_{p}$, respectively. For clarity, the detuning $\Delta \omega_{p}$ is also depicted.

The cascade configuration chosen for the EIT analysis comprises the first three l=0 energy levels and is modeled in Figure 2.

Within the density matrix approach, an expression for the imaginary part of the linear susceptibility can be derived in terms of the probe field and Rabi frequencies as follows [32]:

(7) $$\chi^{\prime \prime }\left(\omega_{p}\right)=\frac{{\left|M_{21}\right|}^{2}\;\sigma_{12}}{\hbar \;\varepsilon_{0}\varepsilon_{r}}\times \frac{\Gamma_{13}\left(\Gamma_{12}\;\Gamma_{13}-\Delta_{p}^{2}\;+\Omega_{c}^{2}/4\right)\;+\;\Delta_{p}^{2}\;\left(\Gamma_{12}\;+\;\Gamma_{13}\right)}{{\left(\Gamma_{12}\;\Gamma_{13}-\Delta_{p}^{2}\;+\Omega_{c}^{2}/4\right)}^{2}\;+\;\Delta_{p}^{2}\;{\left(\Gamma_{12}\;+\;\Gamma_{13}\right)}^{2}}\;,$$

where $\Delta_{p}=\left(\omega_{2}-\omega_{1}\right)-\omega_{p}$ is the detuning between the probe field frequency $\omega_{p}$ and that of the |1〉→|2〉 transition, $M_{ij}={\tilde{M}}_{ij}/e$, with ${\tilde{M}}_{ij}=e\langle \Psi_{i}|\vec{r}\cdot {\hat{u}}_{p}|\Psi_{j}\rangle$ being the electric dipole matrix element for a transition between states |i〉 and |j〉 induced by a light field with polarization vector ${\hat{u}}_{p}$. While the general notation uses indices i,j, the specific transition from the ground state to the first excited state (|1〉→|2〉) is represented by the element ${\tilde{M}}_{21}$, as shown in the formula, $\Omega_{c}$ is the Rabi frequency associated with the aforementioned |1〉→|2〉 transition, $\sigma_{12}$ stands for the population difference between the two involved levels, i.e., it is the net density difference of particles found in level 1 compared to level 2. This term is crucial in describing phenomena like absorption and stimulated emission. Additionally, $\Gamma_{ij}$ are the decay rates for the |i〉→|j〉 transition. The Rabi frequency is taken to be fixed for the material throughout this study. The imaginary part of the linear susceptibility is related to the linear optical absorption, allowing for the EIT calculation as in [32]:

(8) $$\alpha_{EIT}\left(\omega_{p}\right)=\frac{\omega_{p}}{c_{m}}\;\chi^{\prime \prime }\left(\omega_{p}\right)\;,$$

where $c_{m}$ is the speed of light in the medium, $c_{m}=c_{vacuum}/\sqrt{\mu_{r}\varepsilon_{r}}$. Finally, the linear optical absorption coefficient (LOAC) for the transition |1〉→|2〉 can be deduced from Equation (8), subtracting all contributions coming from the third level, i.e., $\Omega_{c}$, $\Gamma_{13}\to 0$.

**Figure 3:**
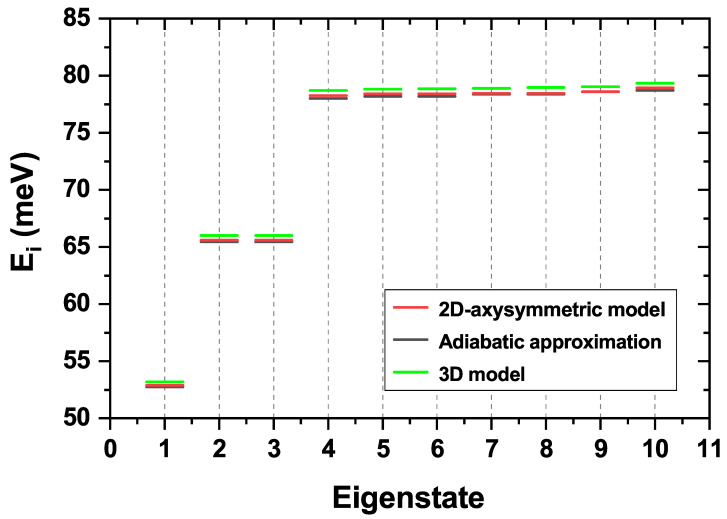
First ten electron eigenenergies in the GaAs/AlGaAs CQDR structure obtained by the 2D-axisymmetric model (red), the adiabatic approximation (black), and the three-dimensional model (green).

To close this section, we provide in Figure 3 the comparison of the first ten electron eigenenergies obtained by solving the three-dimensional Schrödinger equation, by implementing the adiabatic approximation, and through the 2D-axisymmetric model described along this section. The use of the adiabatic approximation is justified by the system’s morphological features, where confined carriers experience significantly greater quantum confinement in the *z*-direction than in the z=0 plane [33]. This geometric asymmetry supports the decoupling of the electron’s motion into two separate kinetic contributions (*z*-axis and xy-plane). The procedure begins by “freezing” the xy-plane motion to solve a one-dimensional Schrödinger equation for the *z*-axis. The resulting eigenvalues are then introduced into the xy-plane Schrödinger equation, reducing the original three-dimensional Hamiltonian to a simplified and numerically solvable two-dimensional problem. As Figure 3 shows, the results from all three methods are in very good agreement, the 3D model being the most precise. Therefore, we can confidently conclude that the implementation of the simpler axisymmetric model is justified for our analysis. At this point, it is worth mentioning that the fact of having seven eigenstates with similar energies does not mean a seven-fold degeneracy, given that the eigenenergies associated with the dot are mixed with those associated with the ring. The latter will be thoroughly discussed later on.

As the comparison in Figure 3 reveals, each model strikes a different balance between accuracy and computational cost. The full 3D simulation serves as our accuracy benchmark; however, its heavy computational demands make it impractical for the extensive parameter sweeps conducted in this study. The adiabatic approximation, while computationally faster, systematically underestimates the eigenenergies, a deviation that grows for higher states where the separability of motion is less valid. The 2D-axisymmetric model, however, proves to be the ideal choice. By leveraging the system’s inherent symmetry, it delivers accuracy that is virtually indistinguishable from the full 3D model but at a fraction of the computational cost. This exceptional blend of precision and efficiency provides a robust justification for its use throughout our analysis.

## 3. Results and Discussion

The Schrödinger equation, given in Equation (6), was solved using the FEM via the COMSOL-Multiphysics software [60] within the effective mass approximation. We computed the lowest confined electron energy states in a GaAs CQDR embedded in an ${\mathrm{Al}}_{x}$G$a_{1-x}$As cylindrical matrix with x=0.3 applying external electric and/or magnetic fields perpendicular to the structure. We calculated the EIT and the LOAC in the presence and absence of the fields mentioned above. The parameters for electrons adopted in the numerical calculations are $m_{\;\mathrm{GaAs}}^{*}=0.067\;m_{0}$, $m_{{\mathrm{Al}}_{0.3}{\mathrm{Ga}}_{0.7}\mathrm{As}}^{*}=0.092\;m_{0}$ (here, $m_{0}$ is the free electron mass) [61] and the barrier height of $V_{0}=262$ meV [62]. For this study, the decay rates and the Rabi frequency are set to $\Gamma_{01}=0.1$ THz, $\Gamma_{02}=5$ THz, and $\Omega_{c}=40$ THz [63]. Calculations were performed for negative and positive values of the electric field *F*. The CQDR profile under study is an adaptation of AFM measurements [25]. In the first stage, the study will focus on analyzing the effects of electric and/or magnetic fields on electron energy levels when the fields are applied perpendicular to the structure.

**Figure 4:**
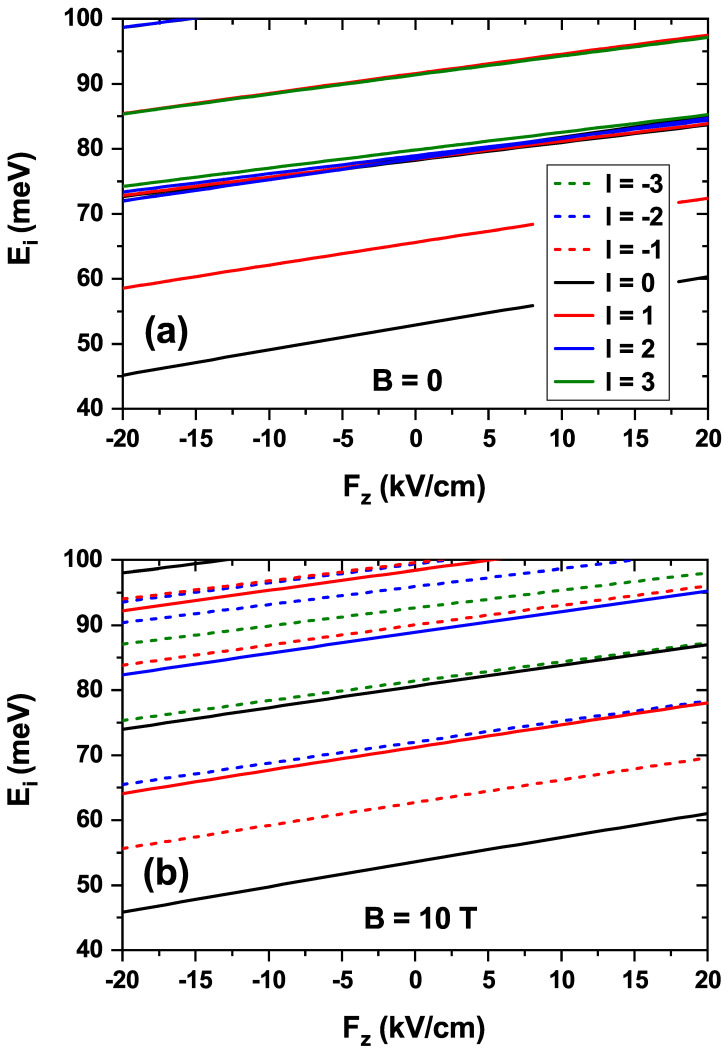
Energy variation of the first low-lying electron states in the absence of magnetic field (**a**) and with an externally applied magnetic field of B=10 T along the *z*-axis (**b**), as a function of the external electric field, applied along the *z*-direction. The negative values of the electric field are related to the negative *z*-axis. In panel (**a**), due to the absence of a magnetic field, states with the same value of |l| are degenerate and therefore the solid and dashed lines overlap.

Figure 4 shows the variation in the electron energies of the first states obtained by applying an electric field directed along the *z*-axis. Negative field values indicate that the field points in the negative *z*-direction. The implementation of a 2D-axisymmetric model allows the distinction of states according to the quantum number *l*. In this work, the first states with |l|=0,1,2,3 are analyzed. Figure 4a is devoted to the case in which no magnetic field is applied, and, therefore, the states with positive (solid lines) and negative (dashed lines) *l* are superimposed, as is straightforward from Equation (6). The energy of the states increases as the electric field shifts from negative to positive values, given that the electron wave function is pushed towards the lower region of the CQDR, becoming more confined as the field intensity increases. The following states, after the first l=0 and |l|=1 states, which correspond to the dot, are concentrated in less than 5 meV. Here, the ring states appear and are intermixed with the dot states. To complete this analysis, Figure 4b shows the energy variation with the electric field in the presence of a magnetic field, also applied along the *z*-direction. This magnetic field is responsible for the degeneracy breaking of the energies associated with l=+1 and l=−1. Comparing Figure 4a,b, states with l≥0 (l<0) are found to display higher (lower) energies than in the absence of the magnetic field. At the same time, all of them show increasing energies as the electric field intensity ranges from more negative to more positive values. In both the absence and the presence of an external magnetic field, a linear increasing trend in the energy levels is observed as the electric field ranges from −20 to +20 kV/cm, such that the levels are practically parallel. A linear fit of these allows the calculation of an average slope, which, in the case of a zero magnetic field, is ≈0.2977 meV cm/kV, while in the case of a 10T magnetic field it is of ≈0.2988 meV cm/kV. The relative standard deviation in the first case is 11.9% and in the second case is 10.7%. The value of this slope can be related to the Stark shift [64,65].

**Figure 5:**
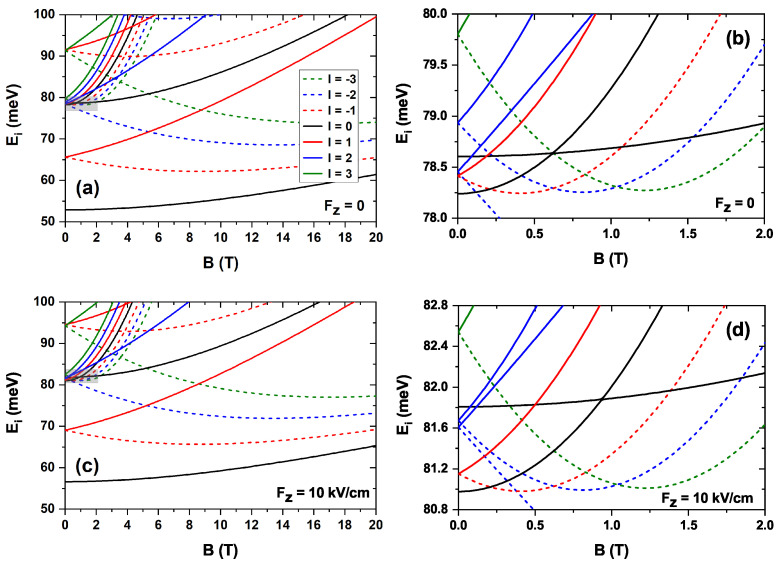
Energy spectrum as a function of the magnetic field intensity for different values of the quantum number *l*. The calculations correspond to zero electric field (**a**) and $F_{z}=10$ kV/cm (**c**). A zoom of the gray-highlighted energy region in (**a**,**c**) for magnetic field values between 0 and 2 T is also presented in panels (**b**) and (**d**), respectively. In all cases, the magnetic field is applied along the positive *z*-direction.

For the sake of completeness, the variation in electron energy is now analyzed in the presence of an externally applied magnetic field, both in the absence and in the presence of an electric field, with both fields parallel to the *z*-axis. The results are shown in Figure 5, which distinguishes the states based on the quantum number *l*, as previously discussed. Both levels whose energy decreases with the magnetic field (typically of QDs) and levels whose energy increases (typically of QRs) are observed. Figure 5a,c show the first electron energies as a function of the magnetic field without an electric field and with an electric field of $F_{z}=10$ kV/cm applied to the nanostructure, respectively. From the comparison between them, it is concluded that the electric field does not alter the main features of the electronic structure, but increases the energies associated with all states. As discussed in Figure 4, the accumulation of states observed corresponds to the emergence of the ring states. Figure 5b,d correspond to a zoom of the shadow region in Figure 5a,c, respectively. The Aharonov–Bohm oscillations, typical of this kind of structure [66], are observed.

**Figure 6:**
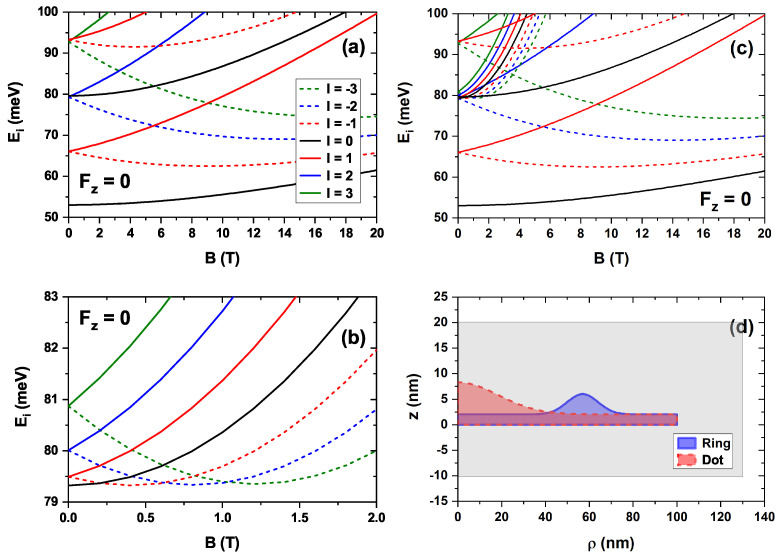
Energy spectrum as a function of the magnetic field intensity for different values of the quantum number *l* as a result of the decomposition of the complete structure in the quantum dot (**a**) and the quantum ring (**b**). The superposition of the results shown in (**a**,**b**) is shown in panel (**c**). A schematic representation of the nanostructures is shown in (**d**). The calculations correspond to a zero electric field. In all cases, the magnetic field is applied along the positive *z*-direction.

The energy spectrum observed in Figure 5 can be better understood by decomposing the CDQR into a colloidal quantum dot (CQD) and a colloidal quantum ring (CQR) with the same wetting layer and dimensions as those shown in Figure 1. The results of this decomposition are shown in Figure 6, where Figure 6a corresponds to the CQD and Figure 6b corresponds to the CQR. The magnetic field sweep is selected according to Figure 5a,c. For simplicity, only the case with no external electric field applied to the nanostructure is analyzed. The superposition of Figure 6a,b is shown in Figure 6c, from which it is clear that the results obtained for the CQDR in Figure 5a,c are consistent with the aforementioned decomposition of the nanostructure, where the coupling between its components causes them to interact and exhibit slight differences. For comparison purposes with previously reported works in the literature, it is recommended to see the results in Figure 1 and Figure 2 of Ref. [67], where the evolution of single-particle eigenenergies in a QD under a parallel and perpendicular applied magnetic field is shown.

The second part of this work is devoted to calculating the EIT in the ladder configuration and LOAC for various values of the electric and magnetic fields.

**Figure 7:**
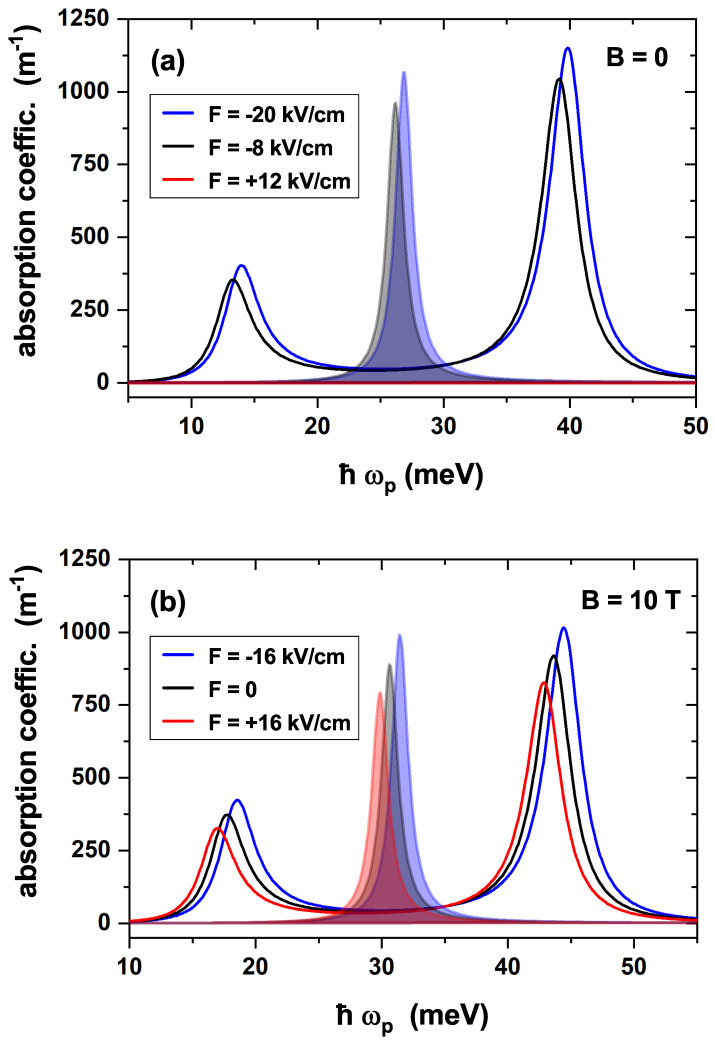
Electromagnetically induced transparency (solid lines) in the ladder configuration associated with the first three l=0 energy levels for three different values of the electric field with zero magnetic field (**a**) and B=10 T (**b**) as a function of the probe field energy. The corresponding values of the linear absorption coefficient in terms of the incident-photon energy are represented by the shaded regions. For visualization, the absorption curves have been divided by 3. In all cases, the magnetic field is applied along the positive *z*-direction.

**Figure 8:**
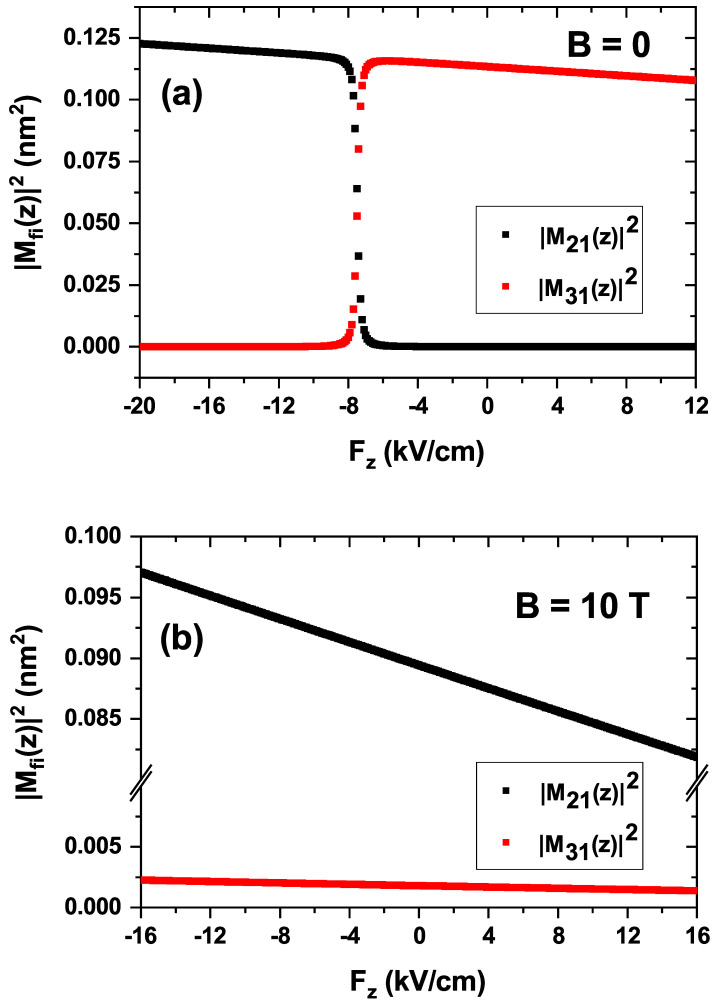
Squared absolute value of reduced ($M_{fi}={\tilde{M}}_{fi}/e$) electric dipole moments involved in the calculation of the electromagnetically induced transparency, $M_{21}$ and $M_{31}$ for zero magnetic field (**a**) and under the effect of a B=10 T magnetic field (**b**), as functions of the externally applied electric field $F_{z}$. The values correspond to the ground (1) and first two l=0 excited (2, 3) states and are obtained for linearly polarized light in the *z*-direction. The magnetic field is applied along the *z*-direction.

To begin with, Figure 7 illustrates the EIT calculation as a function of the probe field energy, $E_{p}$, and the LOAC as a function of the incident photon energy, $E_{ph}$, for three different values of the electric field with and without a magnetic field applied to the nanostructure. Looking at Figure 7a, it is straightforward that the two peaks associated with the EIT present a slightly appreciable blueshift as the absolute value of the electric field increases. Additionally, it is worth noting that the magnitude of the peaks also increases with the absolute value of the electric field. Finally, a particularity arises for the positive value of the electric field, where no EIT is observed. The latter is due to the term of the electric dipole moment matrix corresponding to the transitions involved in the ladder configuration, i.e., between the ground and the first two excited states. This kind of EIT requires an allowed |1〉→|2〉 transition and a nearly suppressed |1〉→|3〉 transition. Figure 6a illustrates how these matrix terms change their behavior at approximately F=−7.5 kV/cm, making zero the EIT for positive *F*-values. The blueshift is justified by the energy levels features shown in Figure 4a. When a magnetic field B=10 T is applied together with the electric field, the EIT in the ladder configuration is observed for positive and negative values of the electric field. This is illustrated in Figure 7b and is justified by the dipole moment matrix terms displayed in Figure 8b. A slight blueshift occurs as the electric field ranges from positive to negative values. On the other hand, shadowed regions in Figure 7a,b show the LOAC as a function of the incident photon energy, which is centered at the minimum located between the two EIT peaks. Again, the term of the zero electric dipole matrix for the transition |1〉→|2〉 at positive electric field values is responsible for the zero LOAC in Figure 7a.

**Figure 9:**
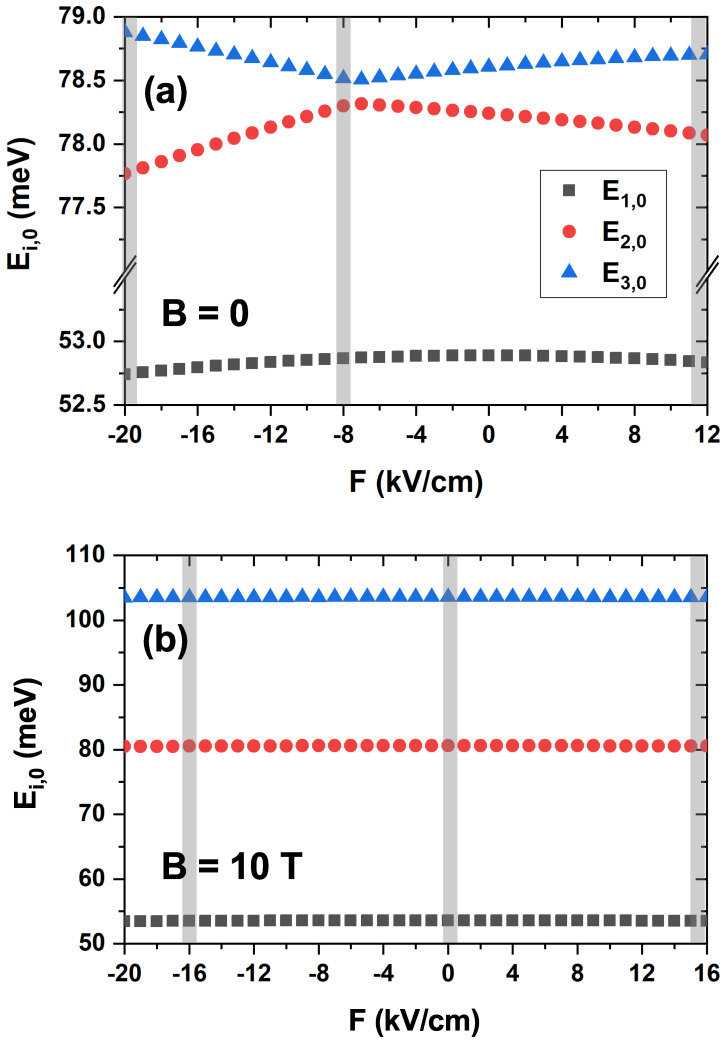
Energy of the first three l=0 states ($E_{1,0}$, $E_{2,0}$, and $E_{3,0}$) as a function of the applied electric field *F*, calculated for (**a**) a zero magnetic field (B=0) and (**b**) a magnetic field of B=10 T. Note that a dominant linear component has been subtracted from the plotted energy values to emphasize the non-linear Stark effect. The gray shaded areas correspond to the electric field values used in Figure 7.

The behavior of the dipole matrix terms shown in Figure 8, which directly impacts the EIT spectra in Figure 7, is governed by the energy shifts of the electronic states under external fields. To illustrate this dependence, Figure 9 plots the energies of the first three states with angular momentum l=0, denoted as $E_{i,l}$ (where i=1,2,3 is the principal quantum number and l=0), as a function of the applied electric field. Figure 9a shows the case without a magnetic field (B = 0 T), while Figure 9b corresponds to a magnetic field (B = 10 T). The gray shaded areas indicate the specific electric field values used for the calculations in Figure 7.

To better visualize the non-linear component of the Stark effect, which is crucial for determining the dipole moments, a dominant linear trend has been subtracted from the energy values. This data processing step enables the clear observation of quadratic shifts and level anti-crossings, which are otherwise obscured, providing direct insight into the physics governing the dipole matrix elements. A linear regression was performed on the data shown in Figure 4 to verify that the energy variation with the electric field was linear. This was done in the absence of a magnetic field and in its presence (B=10T). The slopes of the fitting lines were obtained for the first three levels associated with the quantum number l=0. Figure 9 was obtained as follows:

(9) $$E_{i,0}\left(\mathrm{Figure}\;9\right)=E_{i,0}\left(\mathrm{Figure}\;4\right)-slope\;\times \;F\;\left(\mathrm{kV}/\mathrm{cm}\right)\;.$$

**Table 1:** Slopes obtained from Figure 4 via linear regression for the first three eigenstates.

Eigenstate	Slope (B=0)	Slope (B=10T)
	**(meV·cm/kV)**	**(meV·cm/kV)**
$E_{1,0}$	0.3783	0.3799
$E_{2,0}$	0.2877	0.3267
$E_{3,0}$	0.3114	0.2741

In Table 1, we present the parameters concerning the curves in Figure 9 obtained via Equation (9).

**Figure 10:**
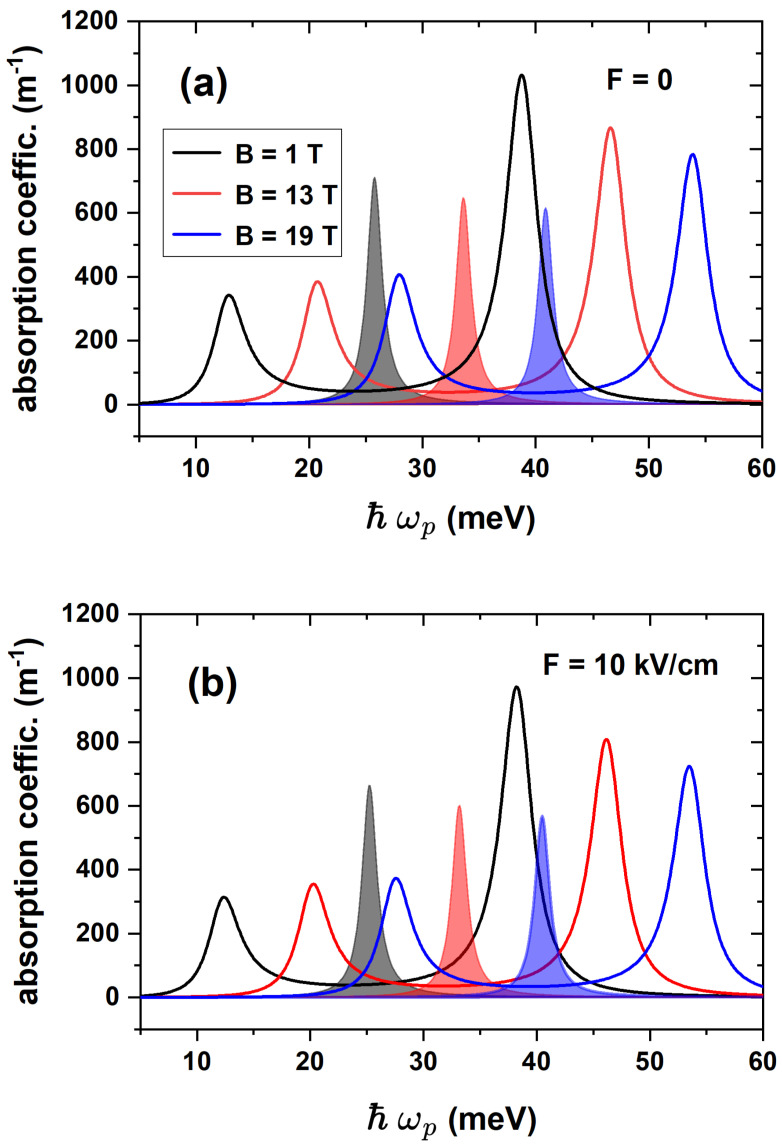
Electromagnetically induced transparency in the cascade configuration and the corresponding values of the linear absorption coefficient associated with the three first l=0 energy levels for three different values of the magnetic field with zero electric field (**a**) and $F_{z}=10$ kV/cm (**b**) in terms of the incident photon energy. In all cases, the magnetic field is applied along the positive *z*-direction.

Figure 10 illustrates the dependence of the EIT and LOAC on the magnetic field, both in the absence and presence of an external electric field. Figure 10a shows the results for a zero electric field, and it is clear that the EIT peaks shift toward higher energies as the magnetic field strength increases and that the LOAC peaks undergo the same energy shift. Figure 10b is homologous to Figure 10a, but subjecting the system to an external electric field of F=10 kV/cm. Comparing the EIT and LOAC peaks in both panels, it is straightforward that the effect of the electric field is practically negligible compared to the magnetic field, with only a slight variation in the magnitude and position of the peaks being observed. The magnitude of the EIT peaks decreases slightly between Figure 10a,b, and the shift of the peaks to lower energies in the presence of the electric field is practically imperceptible. The electric field causes once more the emergence of the LOAC peaks at lower energies and their magnitude to be slightly lower compared to the case without the electric field. All this is justified by the electronic structure shown in Figure 5.

**Figure 11:**
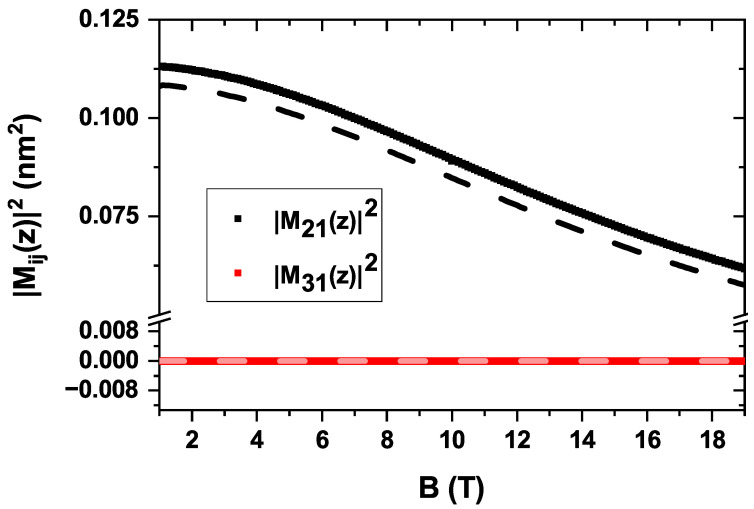
Squared electric dipole matrix elements, $|M_{ij}{|}^{2}$, as a function of the applied magnetic field *B*. The figure shows the elements for the transitions from the ground state (|1〉) to the first two l=0 excited states, |2〉 (black) and |3〉 (red). Solid lines correspond to a zero electric field (F=0), while dashed lines represent the results under an external electric field of $F_{z}=10$ kV/cm. Calculations assume linearly polarized light along the *z*-direction.

In contrast to Figure 7, the non-zero EIT shown in Figure 10 is explained by the behavior of the electric dipole matrix elements, which are depicted in Figure 11. This figure displays the squared dipole moments for the |1〉→|2〉 transition ($M_{21}$) and the |1〉→|3〉 transition ($M_{31}$) as a function of the magnetic field. The results are shown for both a zero electric field (solid lines) and an external electric field of F=10 kV/cm (dashed lines).

A direct comparison between the solid and dashed curves reveals that the influence of the electric field on the dipole moments is minimal, confirming that the system’s optical response is predominantly governed by the magnetic field. The most significant trend observed is the systematic decrease in the primary dipole matrix element, ${\tilde{M}}_{21}$, as the magnetic field strength increases. This behavior is a direct consequence of magnetic confinement. The applied Lorentz force radially compresses the electron wavefunctions, leading to a reduced spatial overlap for the dipole transition. Consequently, the magnitude of ${\tilde{M}}_{21}$ is diminished, which directly suppresses the transition probability and, therefore, the overall absorption intensity [51]. It is also noteworthy that the ${\tilde{M}}_{31}$ element is nearly zero across the entire range, indicating that the |1〉→|3〉 transition is dipole-forbidden for this light polarization, consistent with the selection rules.

## 4. Conclusions

In this study, we have analyzed the electronic structure and optical properties of a GaAs/${\mathrm{Al}}_{0.3}$G$a_{0.7}$As CQDR system under the influence of external electric and magnetic fields. Using a finite element method within the effective mass approximation, we demonstrated that both fields significantly influence the confined electron energy levels, with the magnetic field producing notable Aharonov–Bohm oscillations and a more substantial impact on the energy spectrum than the electric field. These external fields not only shift the electronic states but also modulate the LOAC and EIT behavior, thereby providing mechanisms for dynamic control of the nanostructure’s optoelectronic response.

The EIT phenomenon in a ladder configuration was thoroughly investigated, revealing that its occurrence and intensity are highly sensitive to the direction and magnitude of the applied electric field. In particular, a suppression of EIT was observed for positive electric field values in the absence of a magnetic field, an effect traced to vanishing dipole transition matrix elements. Conversely, applying a magnetic field restored the EIT for all electric-field values, leading to an overall enhancement in the tunability of optical transparency and absorption. These findings highlight the superior role of magnetic fields in precisely modulating light-matter interactions in CQDRs.

Overall, our results demonstrate that CQDR systems are promising platforms for developing tunable optoelectronic devices, with magnetic fields offering a more robust control parameter than electric fields for manipulating EIT and LOAC. These findings contribute to the understanding of quantum interference effects in complex nanostructures, opening pathways for their application in photonic devices and quantum information processing technologies.

Our study thus solidifies the potential of CQDR systems as highly tunable platforms, establishing that magnetic fields, in particular, offer a precise and powerful tool for sculpting their optical response. While this work provides a foundational understanding under idealized conditions, its scope naturally sets the stage for further investigations. For instance, our zero-temperature model offers a clear baseline, from which the next logical step involves incorporating thermal effects, such as phonon interactions, to bridge the gap between these theoretical predictions and the performance expected in experimental setups. Similarly, exploring other tuning parameters, such as hydrostatic pressure, could introduce new dimensions of control, while accounting for the inherent asymmetries of real-world nanostructures would further refine the model’s predictive power. Ultimately, these findings lay the groundwork for future experimental efforts aimed at harnessing quantum interference in these complex nanostructures, potentially unlocking their use in next-generation photonic devices and quantum information technologies.

## Data Availability

All the files with tables, figures, and codes are available. The corresponding author will provide all the files in case they are requested.
